# Factors that affect Israeli paramedics’ decision to quit the profession: a mixed methods study

**DOI:** 10.1186/s13584-019-0346-0

**Published:** 2019-11-01

**Authors:** Keren Dopelt, Oren Wacht, Refael Strugo, Rami Miller, Talma Kushnir

**Affiliations:** 1 Department of Public Health, School of Health Sciences, Ashkelon Academic College, Ashkelon, Israel; 2 Department of Health Systems Management, Faculty of Health Sciences, Ben-Gurion University of the Negev, Beer-Sheva, Israel; 3 Department of Emergency Medicine, Faculty of Health Sciences, Ben-Gurion University of the Negev, P.O.B. 653, 84105 Beer Sheva, Israel; 4 Medical division, Magen David Adom (MDA), Tel Aviv-Yafo, Israel; 5 Department of Psychology, Ariel University, Ariel, Israel; 6 Department of Public Health, Faculty of Health Sciences, Ben-Gurion University of the Negev, Beer-Sheva, Israel

**Keywords:** Paramedics, Emergency medical services, Decision to quit the profession, Work satisfaction, Mixed methods study

## Abstract

**Background:**

The rate of Israeli paramedics leaving the profession has been increasing in recent years: 50% leave the profession in three years, for the most part before retirement. While approximately 2500 paramedics have been trained, only about a third of them are still active.

The number of paramedics per 100,000 in Israel is only 8, compared to around 66 in the US, and in light of the shortage of paramedics it is important to enhance retention rates. The purpose of the study was to examine the factors related to paramedics leaving the profession in Israel.

**Methods:**

1. An online survey was sent to 1000 paramedics via Email. 533 were recruited of whom 200 have left the profession. Questions included demographics, job satisfaction, and reasons for leaving or remaining in the profession.

2. In-depth interviews with 15 paramedics who left the profession.

**Results:**

Out of 1000 emails sent, 533 Paramedics responded, of which 200 paramedics who left the profession responded (73% left five years after completing training and 93% after 10 years). Among these former paramedics, choosing the paramedic profession was based mainly on an idealistic sense of mission and eagerness to help others, yet leaving the paramedic profession was related to extrinsic factors: lack of career options, extensive and strenuous physical demands accompanied by unrewarding salaries, unusually long work hours, and shift work that negatively affected family and personal life.

**Conclusions:**

It seems that work conditions, including the lack of opportunities for promotion, lack of professional prospects, and inappropriate compensation for hard work are crucial factors in the decision to leave.

**Recommendations:**

A joint committee of the Ministries of Health, Justice, and Finance and MDA (Magen David Adom, the national EMS in Israel) should be established for the purpose of improving the conditions and modalities of employment of paramedics and providing appropriate emotional support for paramedics who are exposed daily to work under extreme conditions of stress and human suffering. A joint effort could greatly reduce rates of leaving, training costs, and costs incidental to turnover, as well as increase job satisfaction. Moreover, regulating the profession and expanding the scope of practice to new fields like community paramedicine as part of the EMS service and expanding the scope of physician assistants as an academic profession can create opportunities for advancement and diversity at work that will help retain paramedics in the profession.

## Background

Paramedics are at the forefront of the response to medical emergencies and are usually the first to treat medical emergencies. Within a relatively short time, they must make a diagnosis and provide life-saving interventions. In recent years, there has been increasing awareness globally regarding rates of approximately 10% per year of paramedics quitting the profession [1–3]. Furthermore, in the USA Patterson et al. (2010) [4] determined that recruitment and retention of workers are the two most prominent current problems in emergency medical services (EMS). Recent data from Magen David Adom [5] (MDA, the Israeli EMS), strongly support this conclusion and show that while about 2500 paramedics have been trained in the State of Israel, less than a third of them work currently in the profession. As paramedics are a relatively new profession (since 1979 in Israel) which is physically demanding, most people do not reach retirement age. In Israel, there is no early retirement options for paramedics, unlike military, police and fire department. Wacht (2013) [6] found that the work expectancy of paramedics in Israel is very short: half of them quit the profession after only three years of service – going to other fields of employment in the medical field and other professions. A turnover rate that is not only significantly higher than those found in other countries, but also significantly higher than in other health professions in Israel. For example, the rate of nurses abandoning the profession in Israel is less than 1 % per year [7], and the rate of physicians leaving is about 5.5% per decade [8]. Examining other allied health professions in Israel as an example - 93% of Israeli physiotherapists are still engaged in that profession [9].

In addition to the high turnover rate of the profession, there is a shortage of paramedics in Israel. In comparison, in the United States, there are 66 paramedics per 100,000 people, and their employment growth is the highest in the health sector, reaching 20% in 2000–2005 and rising [10]. In contrast, in Israel, there are only eight active paramedics per 100,000 people [6]. Moreover, the shortage of paramedics is even more pronounced in peripheral areas, where the lack of infrastructure and human resources from all health professions and access to health services are already compromised.

Leaving the paramedic profession is problematic for EMS services because it may lead to human errors and reduced quality of care [4]. Second, it gives rise to many additional costs, including training newcomers. Third, it results in a loss of clinical knowledge and experience. Fourth, it creates an overload of the EMS system and puts greater pressure on the remaining paramedics. The Israeli EMS is already overextended due to the increase in life expectancy, which has led to an increase in the rate of elderly and chronically ill patients and, therefore, to increasing use of emergency services. According to the data published by MDA, the amount of calls almost doubled in the past decade because of an aging population requiring more health care services. The combination of these issues and the high paramedical turnover rates have created a shortage of employees in the pre-hospital emergency system in the State of Israel.

In Israel, paramedics work mainly in pre-hospital ambulances, whereas, in other countries, they also work in hospitals, clinics, and elsewhere. An acute shortage of manpower would likely make it difficult to develop employment opportunities for them in addition to ambulances, since other more varied options may result in them quitting the EMS system. Even so, when paramedics have no options to develop professionally, they may seek other opportunities. Thus, the aim of this study is to examine personal and organizational factors related to the abandonment of the profession by paramedics in Israel.

### Reasons for quitting the profession among paramedics

Turnover decisions, according to Mano-Negrin (2001) [11], are the result of weighing several factors, mainly the employee’s perceived fit between himself or herself and the place of work and the available market alternatives. The smaller the gap between the employee’s personal preferences and the characteristics of the organization, the smaller is the worker’s desire to change his/her workplace. Another factor that affects the worker’s decision is the labor market. The more active the labor market relevant to the employee’s skills is, the greater the number of opportunities that arise for employment, which may lead to greater personal empowerment [12]. Feelings of stress, burnout, and dissatisfaction with work are additional causes of turnover [3, 13, 14]. Indeed, studies conducted in various countries over the years regarding the work of paramedics have found high levels of stress and work overload, leading to high levels of burnout [15–17].

Paramedic work is characterized by a large number of risk factors for burnout: shift work, witnessing the pain and suffering of human beings, exposure to severely upsetting sights, Post-Traumatic Stress Disorder (PTSD), uncertainty, injuries, violence, and more [18, 19]. The levels of stress that these workers experience on the job are even higher than those experienced by physicians in emergency rooms [20, 21]. Paramedics working in ambulances report the highest level of burnout because they treat people who are hovering between life and death, along with sharp transitions from a state of calm to a state of extreme emergency [16, 22]. Johnson et al. (2005) [23] compared the level of stress among 26 types of occupations considered “demanding” (police officers, nurses, firefighters, etc.). They found that paramedics ranked first in the negative effects of work on physical health, fourth in the negative psychological effects (after social workers, teachers, and firefighters), and second in work dissatisfaction (after prison guards). Considering all factors together, paramedics reported the highest levels of stress and workload.

Brown, Dawson & Levine (2003) [1] found that 62% of paramedics in their study reported that they did not have an appropriate retirement plan, and 94% believed they should be compensated more for their work. In addition, they were not satisfied with the attitude of their superiors and felt that they did not have professional promotion opportunities. Revicki & Gershon (1996) [24] examined the relationships between group support, supervisor behavior, work stress, and mental health among paramedics. They reported that group support and positive supervisor behavior were associated negatively with stress at work and positively with mental health. Moreover, there is evidence that support from the supervisor reduces levels of stress, distress, and ultimately, turnover [25, 26].

It appears that quitting the paramedic profession is the result of a complex decision-making process. The high turnover rate among paramedics may be due to a variety of organizational and personal reasons. In Israel, research has not yet been conducted to examine in depth the reasons for quitting this profession and uncovering these factors could help create a policy to reduce turnover. The growing demand for this professional workforce, the already felt shortage of personnel, and the high turnover rate dictated the need for the present study.

### The study objectives

To examine personal and organizational factors related to quitting the paramedic profession in Israel.

## Methods

The study included the use of two types of tools: an online questionnaire and in-depth interviews.

### An online survey among paramedics who had quit the profession

#### The study population and the sample

In Israel, about 2500 paramedics have been trained to date, of whom 650 are still active. In other words, about 1850 (74%) have abandoned the profession. The questionnaire was sent by email to all paramedics (approximately 1000 in number) for whom an updated email address was found. The sampling method used was a convenience sample, and all paramedics who responded positively to the request to participate in the survey were included in the study. The response rate was 53%. Among the 533 participants, 333 were active and the 200 (**37.5%**) who had quit the profession constitute the study sample in this article (there were different questionnaires for each group).

#### Research tool

The online questionnaire was conducted in Hebrew and included demographic details, background information related to the field of work as a paramedic and tools assessing satisfaction with work as a paramedic and reasons for quitting the profession. The last two tools were compiled by the researchers and included items drawn from published studies on job satisfactions and reasons for leaving this profession (e.g. [27–29],). The two tools were validated at face value to be relevant to the work of paramedics in Israel by five experts: two experts in organizational behavior and three senior paramedics. The questionnaire parts are as follows:

1. Demographic details – sex, marital status, age, country of birth, and education.
2. Background information related to the field of work as a paramedic – year of completion of training, place of training, whether the respondent would choose the profession again (yes/no), main field of activity, years of experience as a paramedic, and whether currently he or she volunteered as a paramedic apart from another job (yes/no).
3. Satisfaction with work as a paramedic – The questionnaire included five questions: satisfaction with professional training/preparation, with the ability to make decisions at work, with the ability to save lives, and from the work in general. The answers were recorded on 5-point Likert scale ranging from (1) *very little* to (5) *to a very large extent*. The reliability of the questionnaire is α = 0.70. A high score indicates a high degree of satisfaction.
4. Reasons for quitting the profession: This questionnaire included 8 items on a 7-point Likert response scale, ranging from (1) *very little* to (7) *to a very large extent*. The questionnaire contained statements such as: “I felt emotionally drained by my job”; “I wanted to study another field”; “Shift work conflicts with my family life/relationships”; “The salary was too low/I got a better job offer”; and “I felt that I had no career promotion opportunities.” In addition, the participants were asked (open-ended question) to freely indicate three factors that might have kept them in the profession.

#### Procedure and data collection

The study was approved by the Ethics Committee of the Faculty of Health Sciences at Ben-Gurion University. The sampling frame of paramedics who trained in Israel was received from the MDA, with the survey conducted online during September–November 2014. The data was imported and processed anonymously using SPSS v. 21.0.

### In-depth interviews

#### Population

In the online survey, paramedics were asked to submit a mobile phone number if they had quit the profession and would agree to be interviewed. 74 paramedics provided this contact information, and we contacted about half of them (according to a mix of sex, seniority in the profession, place of training, and geographic location). 15 responders agreed to participate in the interview. In total, 15 in-depth interviews were conducted with 9 men and 6 women, two of whom are currently engaged in nursing, three are medical students, three are students in other fields, four are employed in managerial positions, and three are engaged in medical marketing.

The interviews were conducted face-to-face. One interviewer conducted all the interviews. The interviews were semi-structured. All the interviewees were asked the same questions, and they could add more information freely. The interviews included questions about the reasons for choosing the profession, the training process, how long they worked as paramedics and in what facility, schedule of the shift work, what they liked or disliked about working as a paramedic, reasons for quitting the profession, what they would do to promote the profession, if they would choose the profession again, and their level of satisfaction with their current occupation.

#### Data analysis

Upon the interviewees’ consent, the interviews were recorded and transcribed. The data were then analyzed using the Thematic Analysis method. The analysis included both deductive themes derived from the subject of the study and the literature review, as well as inductive themes that emerged from the data. The analysis was carried out by focusing on thorough and comprehensive acquaintance with the entire data by means of a lateral reading of all the interviews by two researchers, and identification of themes related to the research questions. Both researchers identified similar themes in the interviews.

## Results

### An online survey among paramedics who had quit the profession

#### The sample

As can be seen in Table 1, most of the subjects were men, born in Israel, and who had an academic education. Their average age was 33.8 ± 7.3, and the average number of years since they were certified as paramedics is 9.1 ± 5.3.

**Table 1:** Description of Sample Characteristics (*n* = 200)

Characteristic	n	%
Men	142	71
Born in Israel	164	82
Academic education	162	81
Married or living in a relationship	132	66
Accredited in MDA^a^	70	35
Accredited at Ben-Gurion University	77	39
Accredited in IDF^b^	45	22
Accredited at Assaf Harofeh Hospital	8	4
Age (in years)	33.8 ± 7.3	
Mean years since qualification	9.1 ± 5.3	

^a^*MDA* Magen David Adom, Israeli EMS

^b^*IDF* Israel Defense Forces

#### Professional life expectancy

Participants were asked to indicate how many years they had worked as paramedics since completing training until they quit the profession. Excluding paramedics trained in the IDF for military service, the average was 4.3 years, and the median was 3 years (i.e., half of them left the profession three years after finishing training). The distribution of frequencies was as follows: 42% had worked in the profession up to two years and then retrained in other professions; 31% had worked as paramedics 3–5 years after the end of training, 20% between 6 and 10 years, and 7% worked for 11 years or more before retraining.

#### Choosing to become paramedics and satisfaction with work as paramedics

The participants were asked whether they would choose again to be a paramedic. 20% answered “not at all/very little”, 25% “somewhat”, half (52%) answered “much/very much”, and the rest (3%) did not know**.**

As can be seen in Table 2, the highest satisfaction level was reported regarding the ability to save lives, followed by satisfaction from professional training, equipment required to carry out the work, and the ability to make decisions at work. Only about a quarter (24%) expressed satisfaction with paramedic work in general. About two-thirds (64%) noted that a paramedic’s work integrates well with family life to a very small extent.

**Table 2:** Work Satisfaction as a Paramedic (*n* = 200)

Factor/facet	Large extent (%)	Moderate extent (%)	Little extent (%)	Irrelevant (%)	Mean^a^ ± sd
Ability to save lives	88	5	4	3	0.79 ± 4.49
The training/professional preparation	75	13	10	2	0.91 ± 3.96
The equipment needed to perform the work	69	15	15	1	0.95 ± 3.73
The ability to make decisions at work	56	18	24	2	1.17 ± 3.43
Work as a paramedic in general	24	15	55	6	1.08 ± 3.43
Work integrates well with family life	14	18	64	4	1.28 ± 2.60

^**a**^Mean was calculated excluding “irrelevant” answers

#### Reasons for quitting the profession

As can be seen in Table 3, of 17 possible reasons, the main reason for leaving the profession, in descending order, was the lack of opportunities for professional promotion (83%). The other reasons were poor wages (67%), receiving another job offer with better conditions (60%), the respondent began studying another field (46%), and shift work conflicted with family life (39%). About a third (31%) claimed that they were completely exhausted at the end of the work day. About a quarter (29%) agreed to a large extent that the work was too hard and that the physical conditions were difficult. About 27% were frustrated at work, feeling drained and mentally tired. About a quarter (24%) did not get along with their superiors. A fifth agreed to a large extent with the statement that they had reached the limit, and 14% felt they had become indifferent to the patients, depersonalizing them. Only 6% did not get along well with co-workers.

**Table 3:** Reasons for Quitting the Profession (*n* = 200)

Reasons for leaving	Large extent (%)	Moderate extent (%)	Small extent (%)	Mean ± sd
I felt that I had no career advancement opportunities	83	4	13	1.78 ± 5.60
The wages were too low	67	14	19	1.95 ± 5.08
I got a job offer with better terms	60	8	32	2.25 ± 4.63
I wanted to study another field	46	9	45	2.19 ± 3.79
Working in shifts made life difficult for my family life	39	16	45	2.04 ± 3.66
I felt ashamed of my work	31	19	50	1.95 ± 3.36
The physical working conditions were hard for me	28	19	53	1.97 ± 3.34
I felt I was working too hard	29	17	54	1.90 ± 3.30
I felt exhausted at the end of the work day	31	16	53	1.95 ± 3.25
I felt frustrated by my work	27	13	60	1.90 ± 3.15
I felt emotionally drained by my work	26	16	58	2.01 ± 3.05
I did not get along very well with my superiors	24	10	66	1.90 ± 2.83
I felt as if I had reached the limit of my suffering	20	12	68	1.84 ± 2.62
I felt more indifferent to patients	14	14	72	1.67 ± 2.41
I felt tired every time I had to face another day’s work	25	13	62	1.83 ± 2.97
I felt I was beginning to treat some of my patients as if they were objects	14	14	72	1.68 ± 2.40
I did not get along very well with my coworkers	6	7	87	1.26 ± 1.85

#### What would have kept them in the profession?

The participants were asked in an open-ended question to indicate three things that would have kept them in the profession. 157 participants answered the question, and the answers were encoded in the SPSS program. The responses were as follows: higher wages (78%), promotion opportunities at work (40%), supportive and fair management/fair treatment from management (27%), better employment conditions/better retirement conditions (17%), less stress at work/shift hours (15%), anchoring the profession in legislation (13%), satisfaction and enjoyment from work and ability to save lives (11%), increased authority (11%), the ability to successfully combine work and family life/possibility to work only in the morning hours (10%), appropriate working conditions and additional positions (8%), and interest and intellectual challenge (5%).

### Analysis of in-depth interviews

The analysis of the in-depth interviews revealed five prominent themes: a love for the profession and a sense of mission as providers of job satisfaction and meaning in life, the need for versus the lack of emotional support by management, what the paramedics disliked at work, reasons for quitting the profession, and the advantages and disadvantages of their current occupation compared with being a paramedic.

#### Theme 1: meaningful emotional attachment to the profession: the love of the adrenalin rush, the sense of mission as providing satisfaction and meaning in life

All the interviewees expressed their love for the profession and the sense of mission they felt when they chose to become paramedics. Particularly noticeable was the use of emotional and powerful words to describe the connection to the profession: love, tremendous feelings, addiction, amazing, and adrenalin.

"In my case, it was really an ideology... I was a young man when I enrolled in training ... I wanted to help people and change the world... I loved that it was a compassionate profession. First of all, this profession is meant for people who really have compassion, who want to help; a profession that shows the good and positive sides of society".

(interviewee 13)

"Reaching home at the end of a shift and saying 'I changed something, I did something,' it is a very significant achievement... work in an ambulance as a paramedic is the ultimate gratification, no other job is more rewarding in terms of satisfaction, and immediate results. I do not think there is anything else that gives you the same benefits – at that moment".

(interviewee 4)

"I fell in love with this... the need to make decisions in a short time, to get data, to draw conclusions, when you cannot make an error ... and I really enjoyed it... I really enjoyed the responsibility... I really enjoyed the adrenaline … There are some situations that will accompany me all my life ... You do not forget the girl whose life you saved in a fire, and that those were the two critical seconds that saved her, you will not forget it".

(interviewee 6)

"I think it was an amazing time in my life. I got tremendous satisfaction that I might not have received from almost any other profession".

(interviewee 10)

#### Theme 2: the need for versus the lack of emotional support by management

Some paramedics expressed the need for emotional support from their superiors after exposure to human pain and horrific sights, and lamented the lack of it in their work environment. For example: Interviewee 11 stated:

"Everyone ends up with some kind of Post-Traumatic Stress Disorder, and there's no emotional support in these organizations. What really keeps the people there is the social atmosphere, and there are amazing friendships and people who support one another. In short, we get support but not from management. If the work options were more supportive and more geared towards maintaining the psychological health of paramedics, I would probably have remained in the profession forever".

"Nobody has ever talked to us about this, even after a traumatic event that is a multi-casualty event, which can be very traumatic; and I think that it should be there also after a simple case where you did CPR and did not succeed. Not only in times of war (interviewee 13)".

Interviewee 14 summed up the point: “No one has ever come and asked me, after one event or another... No one has ever asked me if everything was all right with me.”

#### Theme 3: what the paramedics disliked at work

In contrast to love of the profession and a sense of mission, the interviewees raised a number of negative aspects that were repeated in one form or another in most of the interviews, all dealing with the work conditions and lack of promotional options. All the interviewees spoke about the lack of opportunities for advancement and professional diversity/variety.

"I love almost everything about the profession... The main thing I did not like was the lack of opportunity for economic advancement. This is a profession in which it is impossible to make a decent living." (interviewee 15);

"In general, there is no horizon for advancement, no professional horizon, and no respect for professionalism ... At some point, you understand that this is not a place for promoting your career" (interviewee 3);

"I did not like the fact that paramedics can work only in ambulances and cannot work in clinics, hospitals, operating theaters; and I do not like it being a very narrow world, and it does not have to be like that because this is the whole world" (interviewee 13).

Most of the interviewees spoke about the poor wages, shift work, the many hours of work and the conflicts between job and family life that had bothered most of them before leaving the profession.

"You work hours; too many hours and you also want a life ..." (interviewee 6);

"There was a lot of disorder at work. I was unable to know four days in advance when I was going to work and what it would be like. The shifts were constantly changing, the workload was crazy, and the salary was a joke" (interviewee 4).

"I do not think the salary is appropriate. It does not make sense that you spend all the holidays and weekends in the car (ambulance), instead of spending time with the family. The work is physically difficult, there is not enough appreciation, there is not enough authority and independence, and there are no alternatives. You're very fixated on what you're doing".

(interviewee 2)

The physical hardship was also mentioned several times. For example: “I did not like to drag patients from the fourth floor, I did not like to lift equipment to the fourth floor, and I did not like to work without an air conditioner. I did not like it when they threw up in the ambulance” (interviewee 2). “This is a physical job because when you go inside the ambulance and lift a lot of equipment, it gets very difficult” (interviewee 3).

The subject of organizational culture was also mentioned: “I did not like the patronizing by the drivers and the medics, and it upset me and created classes among people” (interviewee 1); “They criticize you, and they never say “well done,“ which is frustrating, I mean, when you do not get positive feedback ... it’s important that the team you work with appreciates you” (interviewee 5).

#### Theme 4: the personal costs of work as reasons for leaving the profession

The reasons for leaving the profession were expressed by the majority of interviewees in terms of the lack of congruence between efforts invested at work and the unreasonable personal and family costs or penalties of staying a paramedic, costs that prevent personal and professional development and “normal” life. This theme presents a clear gap between the demands for investment of great effort in the paramedic job on the one hand, and the lack of adequate compensation on the other, which ultimately led to the decision to quit the profession.

Many interviewees left because of the poor reward for the hard work and long hours of shifts: “Because of the salary and the conditions, the medical staff does everything, takes the patients, and cleans the ambulance. It is weird that for the low pay you also do the work of a cleaner and an orderly. The salary was not worth it” (interviewee 7); “Despite the satisfaction and pleasure, as long as this job is a dead end that does not go anywhere, and on the other hand there is no economic compensation that allows me to ... move forward in life, yes, that is the decision” (Interviewee 4); “I have completed my nursing studies, and this work has good benefits ... social benefits ... not conditions of slavery, my friends work seven days a week, have to do double shifts” (interviewee 1);

Interviewee 15 summed up: “The fact that you do this research and talk about it is because many people quit the profession that does not enable subsistence.”

The penalties to family life accrued by inadequate compensation also contributed to the decision to quit: “I preferred money and preferred life, which means that you have to do night shifts, work on holidays, etc. It is important for me to be at home with the family a little, at least on the Sabbath and holidays” (interviewee 14);

"A day or two after my daughter was born they called me and told me to work a shift ... and it made me realize that if I wanted to be a father and see my daughter growing up, if I worked as a paramedic, it would not happen... The second thing is the price you pay as a person because you work nonstop, you work shifts, you are not at home, so as a bachelor, you are fine, you work 24 hours and you come home in the afternoon, but once you have family the price is too high, you are sacrificing a family here, and that is something that is very, very problematic".

(Interviewee 10).

The most significant reason given for leaving the profession is that despite the many demands and the difficult working conditions, there are no promotion options:

"The job is not rewarding, there are many shifts, and it is hard to manage the job and family life... I would stick to the job if I had something to gain. However, there was nothing. A job that keeps you at the same place with no prospects or opportunities to learn anything else ... to upgrade yourself a bit ... so I had some difficulties with this situation ...

(Interviewee 8).

Today I look at it from the point of view of an adult who thinks about a profession that he chooses for his life, so it is better to choose a profession with opportunities for promotion. If I am not promoted to a managerial position, at least let me advance economically. This is a fantastic profession, but it does not offer you these options.

(Interviewee 15).

Another participant commented: “There are paramedics who are crazy about it. They were youth volunteers in MDA, and they will be paramedics no matter what. But eventually they also reached a glass ceiling that could not be crossed and left” (Interviewee 6).

#### Theme 5: advantages and disadvantages of their current occupation

The advantages noted by the interviewees of their current occupation over work as paramedics reflect exactly the reasons for them leaving the profession into other fields: higher wages, appreciation, promotion opportunities, the possibility of a more comfortable family life, etc. The disadvantages concerned missing the adrenalin rush and the ability to save lives that exist in the work as a paramedic.

Interviewee 8: “The salary is about three times higher. It’s a job that allows you to manage your relationship with your spouse and the family and raise children in a normal way and sleep at night and see friends and enjoy and have hobbies”.

Interviewee 9 clarified:

"This place gives me much better benefits, both financially and in terms of welfare. The benefits of working regular hours. There are also options for promotion".

As to what was missed, interviewee 4 compared:

"There is no comparison for the satisfaction you get from ... this adrenaline and ... working on the ambulance and knowing that you are making a difference to someone. But today, if I compare what I earn per hour, it is ten times more than what I earned as a paramedic, there is no competition".

## Discussion

In this study, we examined the reasons for abandonment of the profession among paramedics in Israel. To this end, we used two complementary methods: an online survey and in-depth interviews with paramedics who had left the profession and undergone retraining in another occupation. It appears that the turnover rate of paramedics in Israel is rapidly increasing. While Nirel et al. (2008) [30] found that this rate was 4% in the first year and 18–21% in five years, in the current study, we found that this rate has increased significantly, to 42% two years after the end of training, 73% five years after training, and 93% within 10 years. Based on the findings of the present study it appears that that the turnover rate among paramedics in Israel is very high. Our findings confirm those found by Wacht (2013) [6].

The most significant reasons for quitting the paramedic job cited by the interviewees in the present study are similar to those mentioned in previous studies in other countries [27, 28]. Moreover, the reasons cited in the qualitative interviews were consistent with those listed in the online survey. The paramedics were firstly concerned with the lack of options for promotion and diversity in the profession, and second, by the low wages. For many, shift work created work-family conflicts and made life difficult for couples and families. Even paramedics who were satisfied with the sense of having a mission felt that their family life was compromised by their work. Many studies provide evidence supporting the negative effects of external stressors on marriage [31].

It has been found that work–family conflicts increase with greater work demands (e.g., work hours, shift work, stress), especially when the job involves physical and emotional sources of stress that characterize paramedical work. The work–home conflict should be taken seriously since this point was also raised by most of the interviewees as a significant reason for quitting the profession. Others felt worn out, frustrated at work, and emotionally drained.

It, therefore, appears that the job of the Israeli paramedic can be characterized as highly stressful due to high work demands, i.e., physical work, working on weekends and holidays, shift work, double shifts, the toll on family life, the high level of professional skills required in medical emergencies, and exposure to traumatic scenes. Yet these demands are not adequately compensated for by either the pay or the work conditions, especially in light of the absence of opportunities for personal and professional development and promotion. In other words, the high costs (required high investment of mental and physical efforts) in the Israeli paramedic job is not appropriately rewarded and may even harm family life. These appear to be the main factors eventually leading to paramedics’ decisions to leave the profession. The findings of early retirement from work support the prediction of the effort–reward theory of occupational stress [32, 33], according to which the mismatch between efforts invested at work and the rewards given in return causes mental and physical stress due to low occupational social status control (uncertainty about the continuity of social functions in the long term), which may lead to ill health [34].

Another major problem mentioned by many of the interviewees concerns the social and legal status of the paramedical profession. The profession is still not anchored in Israeli law, and MDA is perceived as responsible for it (even though MDA lacks the necessary resources to recruit or retain manpower without external support, making it difficult to advance solutions to the manpower shortage).

The negative implications on the EMS system of such significant turnover is clear in terms of economic costs, loss of knowledge, overload, low morale, and reduced quality of service. But, in broad terms, the findings have implications for the entire Israeli healthcare system. While the issue of the manpower shortage in the nursing and medical professions has been on the Israeli public agenda for many years [35, 36], the situation in the paramedic profession has so far been relatively neglected. In other words, while the Ministry of Health, the Civil Service Commission, and the Ministry of Finance have mobilized and allocated considerable resources to deal with the shortage of physicians and nurses, there is no such commitment for paramedics, even in places where there is a severe shortage of personnel. Magen David Adom was even forced to finance its own work benefits for those moving to the periphery.

Since 2016 the Israeli ministry of health has initiated a physician assistant training program that is targeted mostly at veteran paramedics with a bachelor degree [37]. Now at its third class, this program trains physician assistants to work in emergency rooms across Israel. The effect of this program on the paramedic work force is yet unknown. The program gives veteran paramedics a new option to continue clinical work in a different setting than EMS. A long term follow up of the program and its effects on the paramedic workforce is needed to evaluate how it influences the findings of this study.

Further research is needed to examine in depth the emotional side of the paramedic’s work and its effects on occupational stress, burnout, and turnover, significant causes of the extremely high level of quitting this profession in Israel. There is also a need to examine the economic aspects of the turnover, as well as options for the integration of paramedics into additional treatment facilities.

### Study limitations

The survey was conducted online, and paramedics not in contact with their colleagues could therefore not be located and could not respond to the questionnaire. Similarly, paramedics who lacked digital literacy who do not use the computer were unable to participate in this study. Furthermore, a “convenience” sample may have resulted in selection bias, and it is possible that paramedics who a-priori did not relate and identify with the subject and with the study’s goals have ignored the questionnaire altogether.

## Recommendations

Today, Israeli paramedics have almost no options for working in clinical settings beyond giving treatments within ambulances and some urgent care centers. In order to cope with their high levels of frustration, disappointment, and burnout, paramedics are forced to undertake retraining in non-clinical occupations.

Based on the findings in this study, the recommendations aimed at preventing paramedics from quitting the profession involve increasing the scope of practice and opportunities for promotion, increasing pay and incentives, establishing legal regulation, and developing interventions for coping with stress and burnout.

The initiation of training options for new clinical occupations such as community paramedic, Emergency Care Practitioner (ECP) and expansion of the Physician Assistant (PA) program will enable veteran and academically trained paramedics to integrate into new occupations, without losing the knowledge and clinical experience they have accumulated. Community paramedics are becoming more common in many countries in the last ten years. Community paramedicine offers opportunities for veteran paramedics to continue treating patients in a more relaxed environment, with longer patient interactions and better outcomes for patients. This model has been proposed by the Israeli national EMS.

These new training and work options may provide a partial solution to acute personnel problems in the healthcare system.

In addition, it is essential to take steps towards improving the financial remuneration of paramedics, shorten the shifts, offer grants to paramedics willing to work in the periphery, provide additional social benefits, and reduce the retirement age or offer early retirement options, as is customary in some countries (the retirement age in Israel, is 67 for men and 65 for women). In addition, paramedics should be permitted to work in hospitals and clinics at least part time while still working in EMS.

The establishment of an appropriate incentive mechanism in conjunction with the Ministry of Health and the Ministry of Finance will enable the preservation of paramedics in the EMS system and the meeting of manpower staffing standards in the periphery.

In order to preserve women in the profession over time, the possibility of allowing them to work regular hours without shifts should be considered. All the above measures are likely to reduce the levels of stress and burnout at work. But, furthermore, in order to assist workers suffering from high levels of stress and burnout at work, as well as from the traumatic events and sights they are often exposed to, the EMS system should offer emotional support in the form of support groups and mental health consultations. Finally, a professional paramedic law should be legislated.

While all EMS systems experience high turnover rates, Israeli EMS is unique in some ways that may contribute to the high turnover rate of Paramedics. Israel has a national EMS system – a unified system that delivers EMS services all over Israel. As a national system, MDA employees most paramedics in Israel, and regulates the profession. While this situation is mostly beneficial, having one big employer has its limitations. Another unique aspect of Israeli EMS is that a large number of paramedics are trained in the military \ civil service and when they become civilians at the age of 21, they can work in civilian EMS as the training is similar. Having many young people enter the profession at such a stage in life can direct the profession to be very “young” where young people may work in while training for other professions thus reducing the commitment to the paramedic profession. Lastly, the number of calls the national EMS is dealing with in the last 10 years has doubled without doubling the amount of resources (equipment and manpower) thus increasing the workload dramatically. Changing the utilization of paramedics in EMS to lower the workload and stress can potentially help reduce turnover. This can be done by changing the advanced life support and basic life support mix of ambulances or by utilization of community paramedics as part of the EMS response.

## Conclusions

In conclusion, it seems that the paramedics surveyed chose their profession from the outset out of a sense of mission and an idealistic desire to help others and save lives. However, in practice, their work did not offer adequate compensation and lacked an organizational support system, despite being highly stressful and a source for burnout and erosion of family life. This imbalance has led to an extremely high level of turnover. Therefore, it is essential to improve the congruence between the level of efforts demanded for performing their work tasks on the one hand, and on the other, to improve the economic and social benefits such as pay, status, and appreciation that the workplace provides as compensation for their investment, while supporting family life. Such steps towards solving the problems requires a concerted effort by policy makers in the Israeli healthcare system and ministry of health.

## Data Availability

Please contact author for data requests.

## Abbreviations

EMS: Emergency Medical Services
MDA: Magen David Adom, the national EMS in Israel
